# Effects of fructose-containing caloric sweeteners on resting energy expenditure and energy efficiency: a review of human trials

**DOI:** 10.1186/1743-7075-10-54

**Published:** 2013-08-13

**Authors:** Luc Tappy, Leonie Egli, Virgile Lecoultre, Pascal Schneider

**Affiliations:** 1 Department of Physiology, University of Lausanne, Lausanne, Switzerland; 2 Department of Biochemistry, University of Lausanne, Lausanne, Switzerland; 3 Service of Endocrinology, Diabetes and Metabolism, Lausanne University Hospital, Lausanne, Switzerland

**Keywords:** Fructose, Sucrose, Energy expenditure, Thermogenesis, Basal metabolic rate, Energy efficiency, De novo lipogenesis, Gluconeogenesis, Human

## Abstract

Epidemiological studies indicate that the consumption of fructose-containing caloric sweeteners (FCCS: mainly sucrose and high-fructose corn syrup) is associated with obesity. The hypothesis that FCCS plays a causal role in the development of obesity however implies that they would impair energy balance to a larger extent than other nutrients, either by increasing food intake, or by decreasing energy expenditure. We therefore reviewed the literature comparing a) diet-induced thermogenesis (DIT) after ingestion of isocaloric FCCS vs glucose meals, and b) basal metabolic rate (BMR) or c) post-prandial energy expenditure after consuming a high FCCS diet for > 3 days vs basal,weight-maintenance low FCCS diet. Nine studies compared the effects of single isocaloric FCCS and glucose meals on DIT; of them, six studies reported that DIT was significantly higher with FCCS than with glucose, 2 reported a non-significant increase with FCCS, and one reported no difference. The higher DIT with fructose than glucose can be explained by the low energy efficiency associated with fructose metabolism. Five studies compared BMR after consumption of a high FCCS vs a low FCCS diet for > 3 days. Four studies reported no change after 4–7 day on a high FCCS diet, and only one study reported a 7% decrease after 12 week on a high FCCS diet. Three studies compared post-prandial EE after consumption of a high FCCS vs a low FCCS diet for > 3 days, and did not report any significant difference. One study compared 24-EE in subjects fed a weight-maintenance diet and hypercaloric diets with 50% excess energy as fructose, sucrose and glucose during 4 days: 24-EE was increased with all 3 hypercaloric diets, but there was no difference between fructose, sucrose and glucose. We conclude that fructose has lower energy efficiency than glucose. Based on available studies, there is presently no hint that dietary FCCS may decrease EE. Larger, well controlled studies are however needed to assess the longer term effects of FCCS on EE.

## Introduction

Sugar is a dispensable nutrient, which has been present in low amounts in the human diet throughout most of Man’s history. Its consumption however has markedly increased in Europe and North America over the course of the nineteenth and twentieth centuries. In the USA, high-fructose corn syrup (HFCS) has become available since the 1970s, and has in part replaced sugar as a sweetener [1]. Consumption of fructose-containing caloric sweeteners (FCCS), whether as sucrose extracted from cane or beet, or as mixtures of free glucose and fructose as in HFCS, nowadays accounts for about 20% of the average energy intake of the US population [2,3]. Based on the facts a) that the increase in obesity has roughly paralleled the increase in FCCS consumption over the past 50 years [4,5], b) that FCCS can cause metabolic disorders when present in high amounts in rodents’ [6] or primates’ [7] diet, c) that adverse metabolic effects of high sucrose diets has been specifically linked to their fructose component in rodents [8], and d) that fructose is known to be converted into fat to some extent in liver cells [9], it has been proposed, both in the scientific literature and in the lay press, that FCCS represent a threat to metabolic health [10-12].

Obesity results from the deposition of fat in subcutaneous and visceral adipose tissue, occurring usually over several months to years. Given that one kg of body fat contains approximately 8000 kcal [13], total body energy content increases progressively when people gain weight. This obligatorily implies that obesity results of an imbalance between food energy intake and energy expenditure (EE). The hypothesis that fructose, more than the other nutrients present in foods, is mainly responsible for obesity [14], therefore suggests that it disrupts the complex mechanisms regulating body weight and energy balance, either by increasing food intake or by decreasing EE, at least during periods of active weight gain [15]. The aim of this review was therefore to bring together the results of human studies having assessed the effects of pure fructose or FCCS on EE, and to discuss which metabolic pathways may account for differences between FCCS and glucose.

24-hour EE (24-EE) can be partitioned into basal metabolic rate (BMR), adaptive thermogenesis (including diet-induced thermogenesis (DIT) and adaptive changes in resting metabolism occurring in response to nutritional and thermal factors), and energy expended for physical activity [15,16]. Given the lack of data regarding the effects of FCCS on EE during physical activity, this review focused exclusively on BMR and adaptive thermogenesis.

### Literature search and selection of studies

Original articles were searched using the Ovid MEDLINE(R) database, using (“indirect calorimetry” or “metabolic rate” or “energy expenditure” or “thermic effect” or “thermogenesis”) and (“fructose” “or “sucrose” or “sugar” or “HFCS”or “corn syrup”) as search terms, with results limited to ”human”. Relevant articles were selected if they contained original human data including one of the following comparisons:

Comparison of the DIT induced by FCCS and glucose. In this set of articles we included studies in which DIT was measured by indirect calorimetry after ingestion of a single meal containing isocaloric amounts of FCCS and glucose in subjects while on their usual diet. The studies were included only if BMR was measured for at least a 30-min period, and if post-prandial EE was measured for at least 3 hours following meal ingestion.

Comparison of BMR after consumption of a low- and a high-FCCS-diet for 3 days or more.

Comparison of post-prandial EE or 24-EE after consumption of a low- and a high-FCCS-diet for 3 days or more.

The literature search was completed by articles identified by screening the reference lists of these papers and by the authors’ personal knowledge of the literature, yielding a total of 17 original articles [17-33]. Articles reporting exclusively on subjects with specific diseases other than obesity were not included.

### Comparison of DIT after ingestion of a single load of FCCS or glucose

The DIT is defined as the increase in EE following ingestion of a meal in resting subjects under thermoneutral conditions. Nine studies compared DIT after a single fructose or glucose loads (Table 1) [18-25]. Of these, six studies reported an increased DIT of fructose compared to glucose (including 2 in overweight subjects), two studies reported a larger DIT of fructose but which did not reach statistical significance, and one study reported similar DIT with glucose and fructose.

**Table 1:** Comparison of diet-induced thermogenesis (DIT) with FCCS vs isocaloric amounts of glucose

**Study**	**Test meals**	**Participants**	**DIT glucose**	**DIT fructose**	**(DIT fructose/DIT glucose) X 100**	**P value**
			**(% energy content of the meal)**	**(% energy content of the meal)**		
Sharief et al., 1982	5 g sucrose or glucose/kg ideal body weight	6 normal weight M mean age: not provided	2.6	4.0	154	NS
Tappy et al., 1986	75 g pure fructose or glucose	10 normal weight subjects (6M,4F) mean age: 27 y	6.5	10.2	157	<0.05
Simonson et al., 1988	75 g pure fructose or glucose	9 normal weight subjects (5M, 4F) mean age: 25 y	6.0	9.4	157	NS
Simonson et al., 1988	75 g pure fructose or glucose	9 normal weight subjects (5M, 4F) mean age: 61 y	3.4	10.3	303	<0.05
Simonson et al., 1988	75 g pure fructose or glucose	9 obese subjects (2M, 7F) mean age: 60 y	2.6	8.6	331	<0.05
Schwarz et al., 1989	75 g fructose or glucose in a test meal	20 normal weight subjects (10M, 10F) mean age: 23 y (M); 23 y (F)	10.7	12.4	116	<0.01
Schwarz et al., 1992	75 g fructose or glucose added to a meal	10 normal weight F and 13 overweight F mean age: 23 y (normal weight); 26 y (overweight)	8.4	10.2	121	<0.01
Martines et al., 1994	1g fructose or glucose/kg body weight	6 normal weight M mean age: 35 y	10.7	11.2	105	NS
Fukagawa et al., 1995	75 g pure fructose or glucose	8 young, normal weight subjects (6M,2F) mean age: 21 y (M); 20 y (F)	8.1	9.4	116	NS
Fukagawa et al., 1995	75 g pure fructose or glucose	8 older, normal weight subjects (4M, 4F) mean age: 76 y (M); 71 y (F)	6.5	7.7	118	NS
Blaak et al., 1996	75 g pure fructose or glucose	10 young normal weight M mean age: 28 y	8.0	11.1	139	<0.017
Blaak et al., 1996	75 g pure sucrose or glucose	10 young normal weight M mean age: 28 y	8.0	11.4	143	<0.017
Van Gaal et al., 1999	100 g pure fructose or glucose	13 overweight F mean age: not provided	4.5	6.8	152	<0.006
**Mean**			**6.6**	**9.4**	**162**	
**(range)**			**(2.6-10.7)**	**(4–12.4)**	**(105–157)**	

In our usual diet, fructose is mainly consumed with FCCS (sucrose or HFCS). Upon ingestion of FCCS, glucose and fructose are absorbed in roughly equimolar amounts, and blood glucose and insulin increase. The metabolism of fructose from FCCS may therefore differ substantially from that of pure fructose. Only 2 studies compared the DIT of sucrose and glucose. One of these studies reported a 43% larger thermogenesis with sucrose than with glucose in 10 normal weight subjects [24]. The DIT induced by isocaloric fructose was also measured in the same subjects and was similar to that induced by sucrose. In the other study, the DIT with sucrose (recalculated from the figure included in the original paper) was 53% higher than that of glucose, but the difference did not reach significance possibly due to small number of subjects included [17]. Furthermore, two studies have reported an enhanced DIT when fructose was ingested together with a mixed meal, and hence was metabolized together with glucose issued from starch digestion [20,21].

Altogether, the DIT with FCCS exceeded that with glucose by 62% on average, and the difference reached the level of statistical significance in 6 out of 9 studies. There is therefore strong evidence that ingestion of fructose elicits a larger DIT than ingestion of an isocaloric amount of glucose in healthy subjects. Of note, this effect was equally observed in both gender, and in young and older normal-weight subjects. Interestingly, fructose ingestion elicited a larger DIT than glucose in obese and type 2 diabetic patients as well, and hence there is no evidence that fructose-induced thermogenesis is impaired in insulin-resistant subjects [19]. It was also preserved in patients with liver cirrhosis, and hence there is no reason to believe that it would be impaired as a result of non-alcoholic fatty liver disease [22,34].

In the USA, consumption of HFCS has gradually increased between 1970 and 2000, and represents 30–40% total FCCS consumption [1]. We have found no studies that compared the DIT induced by HFCS to that induced by other sweeteners in humans. The fructose content of HFCS (42-55%) differs only slightly from that of sucrose (50%), and the metabolic responses to these two FCCS in humans are very similar [35]. Furthermore, no significant difference was reported when EE was measured by indirect calorimetry in rats fed diets enriched with either sucrose or HFCS [36].

### Mechanisms accounting for DIT

DIT is defined as the increase in resting EE following ingestion of a meal. This increase in EE can be accounted for by three simultaneously occurring processes: a) changes in the efficiency with which nutrients’ energy is transferred to the cells as “available” ATP; b) an increased ATP need to store dietary nutrients; finally c) an activation of the sympathetic nervous system

#### a) Energy efficiency of fructose

The oxidation of nutrients includes some ATP hydrolysis for their initial activation (for example: phosphorylation of glucose to glucose-6-phosphate, or conversion of fatty acid into fatty acyl-CoA). ATP molecules are therefore both consumed and produced upon oxidation of any nutrient, but only ATP produced in excess of those used are actually made available for cellular energy-requiring processes [37]. As an example, when blood glucose is oxidized, it first undergoes glycolysis, where 2 molecules of ATP are used for the synthesis of glucose-6-phosphate and of fructose 1–6 bisphosphate; thereafter, it is converted, first into pyruvate, then into acetyl-CoA which enters the tricarboxylic acid cycle to be degraded to CO_2_, with the concomitant production of reduced co-enzymes NADH and FADH_2_. In this overall conversion of fructose-1,6-bisphosphate to CO_2_, some ATP molecules are generated at the substrate level, while NADH and FADH_2_ subsequently fuel the mitochondrial respiratory chain, where their oxidation is coupled to ATP synthesis. The values initially proposed by Flatt [37] have however to be slightly revised given our present understanding of transmembrane proton transport and of the molecular mechanisms of mitochondrial ATP synthesis [38]. For our calculations, we assumed that ATP synthase, which comprises 10 c-subunits and 3 γ-subunits in yeast, uses 10/3, or 3.33 protons for the synthesis of each molecule of ATP, to which 1 mole of proton should be added for mitochondrial import of inorganic phosphate. The estimation of the number of ATP used, synthesized, and of net ATP gain during glucose oxidation is shown in Additional file 1: Figure S1. This estimation is dependent on several assumptions regarding the use of protons gradient for substrates (phosphate and pyruvate) transport across the mitochondrial membrane, and may have some degree of inaccuracy because of unaccounted loss of protons due to the buffering capacity of mitochondria and leakage across the inner mitochondrial membrane. Based on these calculations, it can be estimated that 2 moles of ATP are used and 29.5 moles ATP are synthesized, corresponding to a net gain of 27.5 moles ATP / mole glucose. Since the initial energy content of one mole glucose is 686 kcal, the energy efficiency of glucose oxidation, ie: the energy cost of ATP gained, can be estimated as 686/27.5, or 24.9 kcal/mole ATP.

Energy efficiency shows substantial variations according to the class of macronutrients used as energy substrate. Blood glucose and fatty acid oxidation requires the use of a small number of ATP molecules, and hence is associated with a large net ATP gain, a low energy cost of ATP gained, and a high energy efficiency. In comparison, reliance on amino-acid catabolism for energy production requires a higher use of ATP for acetyl-CoA synthesis, and hence proceeds at a larger energy cost of ATP synthesis and a lower energy efficiency [37,39]. During nutrient deprivation, or in subjects placed on a ketogenic diet, gluconeogenesis from amino acids is stimulated to ensure glucose production for the brain; since amino-acid conversion into glucose is an energy-requiring process which uses a substantial amount of ATP, this leads to a lower energy efficiency, which has been proposed to contribute to the effectiveness of ketogenic diets for weight loss [37].

Fructose, although it contains as much energy per molecule as glucose, is not used directly as an energy substrate by extrahepatic cells, which do not express the key enzymes required for its initial catabolic steps. Most cells of the human organism indeed express only the enzymes required for oxidation of glucose and fat, and the liver works as a metabolic plant to metabolize other, less usual nutrients, such as galactose, alcohol, the bulk of amino acids, and fructose [40-42]. The liver, the gut, and the kidney however synthesize substantial amounts of fructokinase and aldolase B, and hence can metabolize fructose to fructose-1-P, and triose-phosphate [42]. Close to the totality of dietary fructose appears to be taken up by the gut and the liver, where it is converted into other energy substrates readily metabolized by extrahepatic cells, such as glucose, lactate, and fat. Although the kidney can metabolize significant amounts of intravenously administered fructose [43], its contribution to the metabolism of dietary fructose is most likely small, since the splanchnic release of fructose is very low (only about 7% fructose appears in the systemic circulation over the 2 hours following ingestion of a 75 grams fructose load) and peak blood fructose concentrations only reach about 0.6 mmol/L [44].

**Figure 1:**
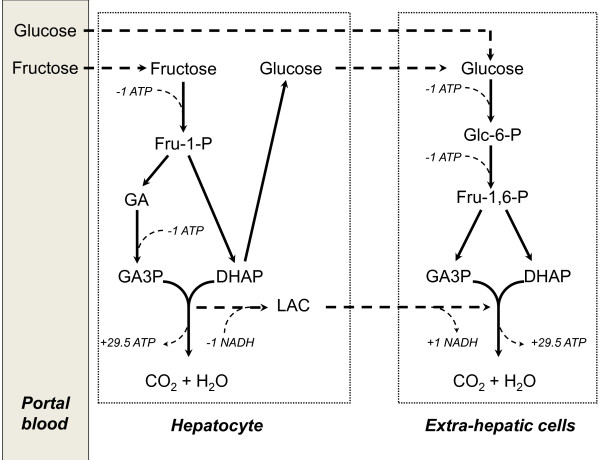
**Metabolic steps accounting for a lower efficiency of fructose compared to glucose.** Fructose conversion into glucose in the liver, followed by glucose oxidation in extrahepatic cells requires the use an additional 2 ATPs compared to the direct oxidation of blood glucose; this is associated with a higher ATP used/ATP synthesized ratio, and thus to a higher energy cost of net ATP gained. F-1-P: fructose-1-phosphate; G-6-P: glucose-6-phosphate; DHAP: dihydroxyacetone-phosphate; GAP: glyceraldehyde-phosphate; GAH: glyceraldehyde; LAC: lactic acid.

After ingestion of a pure fructose load, approximately 50% of the fructose carbon is released as glucose within 4–6 hours in healthy humans [45-47] and up to 15% can be stored as hepatic glycogen [48]. Fructose ingestion also increases blood lactate concentration, indicating that part of the fructose carbons are released as lactate into the bloodstream to be used as an energy substrate by extrahepatic cells. When fructose is oxidized as lactate in extrahepatic cells, the overall number of ATP used (2ATP) and synthesized (29.5ATP) is the same as for glucose oxidation, and the overall energy efficiency is therefore similar to that of glucose. However, 2ATP are used in the liver while 29.5 ATP are synthesized in extrahepatic cells (Figure 1). Lactate oxidation represents the major pathway for fructose oxidation after ingestion of a mixture of fructose and glucose during exercise [49], and may therefore be energetically advantageous for working muscles.

In contrast, when fructose is released as blood glucose to be oxidized in extrahepatic cells, two ATP are used in the liver for glucose synthesis, and two ATP are used in extrahepatic cells, which brings the number of ATP used to 4, while the number of ATP synthesized remains 29.5 (Figure 1). As a consequence, the energy cost of ATP gained increases to 26.9 kcal/mole. This corresponds to an 8% increase compared to glucose.

The low energy efficiency associated with the oxidation of glucose synthesized from fructose makes major contribution to fructose-induced DIT since approximately 40-50% of pure fructose load is converted into glucose and released into the systemic circulation within 6 hours of ingestion [45,47]. The proportion of fructose released as glucose after ingestion of sucrose or mixtures of fructose and glucose remains however unknown.

#### b) Energy cost of storing fructose’s energy within the body

Hepatic glycogen synthesis from fructose requires the hydrolysis of 3 molecules of ATP for each glycosyl unit incorporated into glycogen (one for the synthesis of fructose-1-P, one for the synthesis of 2 glyceraldehyde-3-P (GA3P), and one for synthesis of uridyl-diphosphoglucose (UDPG). Extrahepatic glycogen synthesis from fructose requires one additional ATP for converting blood glucose into glucose-6-P. The ADP produced in this process need be regenerated to ATP, and this increase in EE expenditure contributes to DIT. The theoretical energy cost of nutrients’storage can be calculated as the energy used as ATP (24.9 kcal/mole ATP) relative to the amount of energy stored.

1 mole fructose (686 kcal) + 74.7 kcal from 3 moles ATP used → 1 mole glycosyl units in hepatic glycogen (686 kcal); associated thermogenesis = 10.9%.

1 mole fructose (686 kcal) + 99.6 kcal from 4 moles ATP used → 1 mole glycosyl units in muscular glycogen (686 kcal); associated thermogenesis = 14.5%.

The thermogenesis associated with hepatic and muscle glycogen synthesis would be even larger (respectively 18.0 and 21.8% if gluconeogenesis proceeded from pyruvate instead of GA3P).

Substantial amounts of fructose can be converted into fat in overfed rodents [50]. In humans, stimulation of de novo lipogenesis (DNL) and accretion of newly-synthetized fat can be observed during massive carbohydrate overfeeding [51]. Both glucose and fructose can be metabolized to fatty acids in the DNL pathway, but at a high energy cost (Additional file 2: Figure S2):

12 moles fructose or glucose (8232 kcal) + 2 moles glucose (1372 kcal) + 228 kcal from 9.15 moles ATP used **→** 1 mole triglyceride palmitate (7510 kcal); associated thermogenesis = 31%.

The energy cost, is identical for fat synthesis from fructose and glucose. It is however well documented that hepatic DNL is quantitatively more important, and hence may make a larger contribution to DIT with fructose than glucose [9].

Storing fructose or glucose as fat is highly inefficient compared to storing dietary lipids, which proceeds at a very low energy cost. Conversion of 20% of 75 g fructose into fat over 4 hours post-prandial would indeed increase resting EE by 0.03-0.04 kcal/min, ie similar to the 0.03-0.05 kcal/min post-prandial resting EE difference generally observed after ingestion of fructose vs glucose. Several studies have reported that fructose ingestion increases fractional hepatic DNL up to ten fold in humans [9,52-55]. This represents the relative contribution of DNL to the pool of triglycerides present on VLDL, but provides no information on how much fructose is converted into fat. In one study, the amount of newly synthesized fatty acids secreted as VLDL-triglycerides was calculated in a group of women overfed with fructose during 4 days [56]. Although hepatic DNL increases after a few days on a high fructose diet [33] and the daily carbohydrate intake of the participants was very high (360–390 g/day), they reported that only 5–8 g/day fat were synthetized and secreted as VLDL-triglycerides [56]. This corroborates that only a minor portion of dietary fructose is converted into fat in human.

#### c) Effect of fructose on the sympathetic nervous system

In addition to changes in energy efficiency of substrate utilization related to metabolism of ingested nutrients, part of DIT has been shown to be mediated by a post-prandial stimulation of the sympathetic nervous system [57]. As for glucose metabolism, there is evidence that part of fructose-induced DIT is mediated by a stimulation of the sympathetic nervous system. Administration of a beta-adrenergic antagonist indeed significantly blunted the increase in EE induced by oral [18] or intravenous [58] fructose. Muscle sympathetic nerve activity, which is increased in response to glucose, was however not stimulated by fructose [59]. This suggests that fructose-induced sympathetic stimulation is targeted to other, yet unidentified tissues. The functional significance of sympathetically mediated thermogenesis, and its underlying mechanisms remains unknown.

### Effects of high-FCCS diets consumed during more than 3 days on BMR

Changes in dietary energy, carbohydrate or fat content can alter BMR within a few days, through mechanisms related, at least in part, to changes in sympathetic nervous system activity and in thyroid hormones concentrations [60]. It may therefore be hypothesized that changes in dietary fructose may induce adaptive changes in BMR.

Studies having compared the effects of low- or high- FCCS intakes during 3 or more days on BMR are summarized in Table 2. Data for isocaloric intervention with glucose are also included when available. Cox and collaborators studied two groups of overweight patients fed 25% of their total energy requirements as fructose or glucose drinks over a period of 10 weeks [31,52]. Drinks were added to an ad libitum diet during an initial eight-week period, and resulted in a significant weight gain in both groups. This initial, hypercaloric period was followed by a 5-14-day period during which dietary intakes were adjusted to match energy requirements. BMR decreased by 7% with fructose, but not with glucose. There was no statistically significant difference when the effects of glucose and fructose were compared by a multivariate analysis. McDewitt et al. [26] studied normal weight and overweight women on 4 occasions, each time during 4 consecutive days on either a weight maintenance diet or hypercaloric diets supplemented with 50% excess energy as glucose, fructose or sucrose. They did not report any significant changes in BMR with these interventions. Lê et al. [27,29] and Abdel-Sayed et al. [28] measured the effects of overfeeding with 1.5 or 3 g fructose/kg body weight per day during 1 to 4 weeks, and did not observe any significant change in BMR compared to a weight-maintenance diet. Ngo-Sock et al. [30] compared the effects of a 7-day supplementation with 3.5 g/kg fat-free mass/day fructose and glucose drinks in normal weight subjects, and did not observe any significant changes in BMR compared to baseline with either sugar (Table 2).

**Table 2:** Comparison of BMR after consumption of a high FCCS or high glucose diet for > 3days vs after consumption of a weight-maintenance diet

**Study**	**Dietary intervention**	**Participants**	**BMR before intervention**	**BMR after intervention**	**% change**	**P**	**P**
			**(kcal/min)**	**(kcal/min)**		**HFCCS vs before**	**HFCSS vs high glucose diet**
McDewitt et al., 2000	50% excess energy as fructose during 4 days	8 normal weight F; mean age: 53 y	0.96	0.98	2.60	NS	NS
McDewitt et al., 2000	50% excess energy as fructose during 4 days	5 obese; mean age: 52 y	1.06	1.07	0.80	NS	NS
McDewitt et al., 2000	50% excess energy as sucrose during 4 days	8 normal weight F mean age: 53 y	0.96	0.96	−0.02	NS	NS
McDewitt et al., 2000	50% excess energy as sucrose during 4 days	5 obese F; mean age: 52 y	1.06	1.06	−0.16	NS	NS
McDewitt et al., 2000	50% excess energy as glucose during 4 days	8 normal weight F; mean age: 53 y	0.96	1.00	3.95	NS	
Le et al., 2006	1.5 g fructose/kg/day in excess energy requirements for 4 week	7 normal weight M; mean age: 25 y	1.02	0.98	−3.92	NS	
Abdel-Sayed, 2008	3 g fructose/kg/day in excess of energy requirement during 7 days	6 normal weight M; mean age: 25 y	0.93	0.93	0.00	NS	
Ngo-Sock et al., 2010	3 g fructose/kg/day in excess of energy requirement during 7 days	11 normal weight M; mean age: 25 y	0.99	1.00	0.97	NS	
Ngo-Sock et al., 2010	3 g glucose/kg/day in excess of energy requirement during 7 days	11 normal weight M; mean age: 25 y	0.99	1.01	2.17	NS	
Lê et al., 2009	3 g fructose/day in excess energy requirements for 7 days	8 normal weight M; mean age: 24 y	0.95	0.95	0.00	NS	
Lê et al., 2009	3 g fructose/day in excess energy requirements for 7 days	16 normal weight M with family history of type 2 diabetes; mean age: 25 y	0.98	0.99	1.02	NS	
Cox et al., 2011	25% total energy as fructose, added to an ad-libitum diet during 8 weeks, then 25%, weight-maintenance diet for 5–14 days	9 overweight M and 7 overweight F mean age: 52 y (M); 53 y (F)	1.19	1.10	−7.56	<0.05	NS
Cox et al., 2011	25% total energy as glucose, added to an ad-libitum diet during 8 weeks, then 25%, weight-maintenance diet for 5–14 days	9 overweight M and 7 overweight F mean age: 54 y (M); 56 y (F)	1.17	1.15	−1.71	NS	
**Mean**					−0.02		
**SD**					2.50		

### Long term effects of FCCS on DIT and on 24-hour energy expenditure

To our knowledge, no study actually assessed DIT by continuously monitoring post-prandial EE over several hours in subjects fed a high FCCS diet over several days. Studies having compared the effects of low- or high- FCCS intakes during 3 or more days on post-prandial EE are summarized in Table 3. Data for isocaloric intervention with glucose are also included when available. Cox et al. measured BMR (Table 2) and post-prandial, resting EE over 14 hours after ingestion of fructose-containing meals in overweight subjects before and after a 10-week high fructose diet. They did not observe any significant difference in post-prandial EE (Table 3). DIT was not calculated, but may have been increased with the high-fructose diet, since BMR was reported as decreased in this condition. Theytaz et al. [32] studied normal-weight subjects after 7 days on a low FCCS diet and after 7 days on a hyper-energetic diet with 30% extra-energy as fructose. At the end of each diet, subjects ingested an oral fructose load every hour for 9 consecutive hours; their resting EE was measured during the last 3 hours of this experiment and was not different after the high fructose diet than after the low FCCS diet. Egli et al. [33] performed the same experiment in normal weight subjects after 4 days on a weight-maintenance, low FCCS diet and after 4 days on an isocaloric diet in which 30% fructose substituted starch, and did not observe any difference between the two diets (Table 3).

**Table 3:** Comparison of post-prandial energy expenditure after consumption of a high FCCS- or high fructose-diet vs after consumption of a weight-maintenance baseline diet

**Study**	**Dietary intervention**	**Participants**	**Post-prandial energy expenditure (kcal/min)**		**% change**	**P value**
			**Before**	**After**		
Cox et al., 2011	25% total energy as fructose, added to an ad-libitum diet during 8 weeks, then 25%, weight-maintenance diet for 5–14 days	9 overweight M and 7 overweight females mean age: 52 y (M); 53 y (F)	1.41	1.37	−2.84	NS
Cox et al., 2011	25% total energy as glucose, added to an ad-libitum diet during 8 weeks, then 25%, weight-maintenance diet for 5–14 days	9 overweight M and 7 overweight females mean age: 54 y (M); 56 y (F)	1.40	1.36	−2.86	NS
Theytaz et al., 2012	3.0 g fructose/kg/day in excess energy requirements for 7 days	9 normal weight M mean age: 23 y	0.98	1.06	8.16	NS
Egli et al., 2013	weight-maintenance diet with 30% fructose for 4 days	8 normal weight M mean age: 22 y	0.93	0.96	3.23	NS

We found only one study in which 24-hour EE was measured after 4 days on high FCCS diets. In this study, McDewitt et al. [26] measured 24-hour EE of normal weight and overweight women during 4 consecutive days in a respiratory chamber. Each subject was studied while consuming a weight maintenance diet, an hypercaloric high fructose diet, an hypercaloric high sucrose diet, and an hypercaloric high glucose diet. Compared to the weight maintenance diet, all 3 hypercaloric diets significantly increased 24-hour EE; there was however no difference between the three diets.

### Are the effects of overfeeding with FCCS different from those of overfeeding with other nutrients?

Most studies having assessed the effects of FCCS on BMR were performed over 4–7 days, and failed to document any significant changes in response to excess FCCS. Similar studies assessing the effects of short-term overfeeding with comparable excess energy as fat [61,62] or fat + protein [61] did not report any change in BMR either. This suggests that, in the short term, the effects of excess FCCS are not different from those of other nutrients.

The study by Cox et al. [31,52] surprisingly reports that BMR decreased in overweight subjects who significantly gained weight while consuming a high fructose diet. In that, it markedly differs from shorter fructose overfeeding studies in normal weight and overweight subjects (Table 2). It also differs from several overfeeding studies, in which subjects overfed with 700 kcal fat /day [63], or with about 1000 kcal/day of a mixed diet [64-66] showed either no change or an increase in BMR. The study by Cox et al. [31,52] however had a very specific design, which makes it difficult to compare with other studies. First, participants were fed an ad libitum diet supplemented with fructose during an initial 8-weeks period, but their food intake was not monitored, and therefore their energy overload cannot be quantitatively assessed. Second, participants had their BMR measured after 5 to 14 days during which they were fed a weight maintenance diet. It is therefore possible that part of this discrepancy was accounted for by differences in experimental design between studies. Of interest, the effects of fructose- and glucose-supplemented diets on BMR were not statistically different in this particular study [31], which therefore does not provide conclusive evidence that FCCS exerts different effects on BMR than other sugars.

### Limitations to this review

This review has some limitations which need to be considered before reaching definitive conclusions. First, all the studies retrieved from the literature measured EE by indirect calorimetry; calculation of EE and of net substrate oxidation relies on both total oxygen uptake and respiratory exchange ratio, and on the use of pre-determined stoichiometries for the oxidation of glucose, fat and protein; although respiratory exchange ratio, and calculation of net substrate oxidation may bear a large degree of error under some conditions, calculations of EE remains quite robust [67]. Second, it is possible that there was a bias due to under-reporting of studies showing no differences between FCCS and glucose. Third, and most important, there were a fair number of studies reporting the acute effects of pure FCCS vs glucose loads, but relatively few studies in which FCCS or glucose were included in a mixed meals. Of particular importance, no study was specifically designed to assess dietary-induced thermogenesis in subjects consuming low or high FCCS diets. Only one study compared 24-hour EE of normal weight and overweight women after a weight maintenance diet or a diet supplemented with 50% excess energy as glucose, fructose or sucrose, and observed that it was increased to the same extent with all three sugars [26]. It is therefore difficult to extrapolate the data reported in the literature to real life conditions.

## Conclusions

The data reported in the literature indicate that fructose elicits a larger DIT than glucose, due to a low energy efficiency when fructose is oxidized after having been converted into glucose or fat. The low energy efficiency of fructose is certainly not a causal factor for weight gain, and may even limit energy storage during fructose overfeeding. Of special relevance, de novo fatty acid synthesis from fructose is much less energy-efficient than storing dietary fat, and hence fructose-induced DNL is unlikely to promote weight gain. There is also no solid observation to suggest that the consumption of a high FCCS diet over several days may cause an adaptive decrease of resting EE. Altogether, there is no evidence that FCCS may decrease EE, and body weight gain is to be expected only with hypercaloric high FCCS- diet [68]. These conclusions rest on a limited number of studies including small number of subjects, and larger sized, well controlled studies are clearly needed to evaluate the long term effects of FCCS on EE.

EE represents only one side of the energy balance equation, and there is concern that FCCS may primarily promote obesity by increasing food intake. A recent meta-analysis of intervention trials involving experimental changes in FCCS-containing beverages concluded that changes in body weight were closely related with changes in total energy intake [69]. In other words, a supplementation with FCCS increases body weight only when it is incompletely compensated by a decrease intake of calories from other sources. This further demonstrates that body weight gain results from an increased energy intake rather than a decreased EE, and calls for further evaluation of FCCS effects on food intake.

## Abbreviations

BMR: basal metabolic rate; DIT: diet-induced thermogenesis; DNL: de novo lipogenesis; EE: energy expenditure; 24-EE: 24-hour energy expenditure; FCCS: fructose-containing caloric sweeteners; HFCS: high fructose corn syrup; GA3P: glyceraldehyde-3-P; UDPG: uridyl-diphosphoglucose.

## Competing interests

L Tappy has received research support from Nestle SA and from Ajinomoto Inc. The other authors declare that they have no competing interests.

## Authors’ contribution

LT searched the literature, selected relevant studies, and drafted the manuscript. PS calculated the ATP fluxes involved in metabolic pathways and wrote the related parts of the manuscript. LE and VL verified the literature search and the data reported, and LE, VL and PS critically reviewed and improved the manuscript. All authors read and approved the final manuscript.

## Supplementary Material

Additional file 1: Figure S1 Energy cost of available ATP during oxidation of blood glucose. The diagram on the left depicts the key metabolic steps at which ATP, NADH and FADH_2_ are used or synthesized during oxidation of glucose in extra-hepatic cells. The box on the right part of the figure summarizes ATP used (with negative sign) and synthesized (with positive sign) (left column), cytosolic and mitochondrial NADH and FADH_2_ synthesis (three central columns), and total H^+^ pumped across the mitochondrial membrane (right column). The legend on the left indicates the metabolic pathway where ATP synthesis or NADH/FADH_2_ are generated. At the bottom of the box, the total number of ATP generated at the level of substrate (ie: ATP synthesized–ATP used), and in the mitochondria (calculated assuming that 4.33 H^+^ are needed for the synthesis of each ATP) are indicated. The number of available ATP molecules produced in this process, and the global energy cost of synthesizing one mole of ATP are indicated below the box. Abbreviations; Glc = glucose, Pyr = pyruvate, Q: coenzyme Q, ETC: electron transport chain; Pyr/H+: pyruvate transport; PDH: pyruvate dehydrogenase complex; TCA: tricarboxylic acid cycle. Click here for file

Additional file 2: Figure S2 Energy cost of available ATP when fructose is converted into triglyceride palmitate in adipose tissue, and with subsequent release and oxidation of palmitate. The diagram depicts the key metabolic steps at which ATP, NADH and FADH_2_ are used or synthesized during fructose conversion into palmitic acid in hepatic cells, stored as triglyceride palmitate (with glycerol-3-P obtained from glycolysis) in adipose tissue, and subsequently released as palmitic acid to be oxidized in extra-hepatic cells. Calculations take into account that glycerol released from adipose tissue during lipolysis is reconverted into glucose in liver cells. The box at the bottom right summarizes the ATP used, synthesized, and available ATP made as in Additional file 1: Figure S1. Abbreviations: DHAP: dihydroxyacetone-P; Gly3P: glycerol-3-P; C16:0: palmitic acid; OA: oxaloacetate; Mal: malate; ACS: acyl-CoA synthetase; ACC: acetyl-CoA carboxylase; Cit lyase: citrate lyase; Pyr carb: pyruvate carboxylase; GlyK: glycerol kinase; EHC: extrahepatic cells; Ad: adipocytes; Li: liver; otherwise same as in Additional file 1: Figure S1. Click here for file

## Aknowledgments

The present work, and previous related studies reviewed in this article, have been supported by SNF grants 320030–135782 and 320030–138428 to LT. PS is supported by SNF grant 31003A-138065. The authors wish to express their gratitude to Dr Keith Frayn, from the Centre for Diabetes Research, Oxford University, UK, for having reviewed and criticized a preliminary version of the manuscript.
