# Supplementary material for: Effects of fructose-containing caloric sweeteners on resting energy expenditure and energy efficiency: a review of human trials
Source: Nutr Metab (Lond). 2013 Aug 13;10:54. doi: 10.1186/1743-7075-10-54 (PMC3751443; doi:10.1186/1743-7075-10-54)
Supplement: Additional file 1: Figure S1 — Energy cost of available ATP during oxidation of blood glucose. The diagram on the left depicts the key metabolic steps at which ATP, NADH and FADH2 are used or synthesized during oxidation of glucose in extra-hepatic cells. The box on the right part of the figure summarizes ATP used (with negative sign) and synthesized (with positive sign) (left column), cytosolic and mitochondrial NADH and FADH2 synthesis (three central columns), and total H+ pumped across the mitochondrial membrane (right column). The legend on the left indicates the metabolic pathway where ATP synthesis or NADH/FADH2 are generated. At the bottom of the box, the total number of ATP generated at the level of substrate (ie: ATP synthesized–ATP used), and in the mitochondria (calculated assuming that 4.33 H+ are needed for the synthesis of each ATP) are indicated. The number of available ATP molecules produced in this process, and the global energy cost of synthesizing one mole of ATP are indicated below the box. Abbreviations; Glc = glucose, Pyr = pyruvate, Q: coenzyme Q, ETC: electron transport chain; Pyr/H+: pyruvate transport; PDH: pyruvate dehydrogenase complex; TCA: tricarboxylic acid cycle. [file 1743-7075-10-54-S1.ppt]

## Slide 1
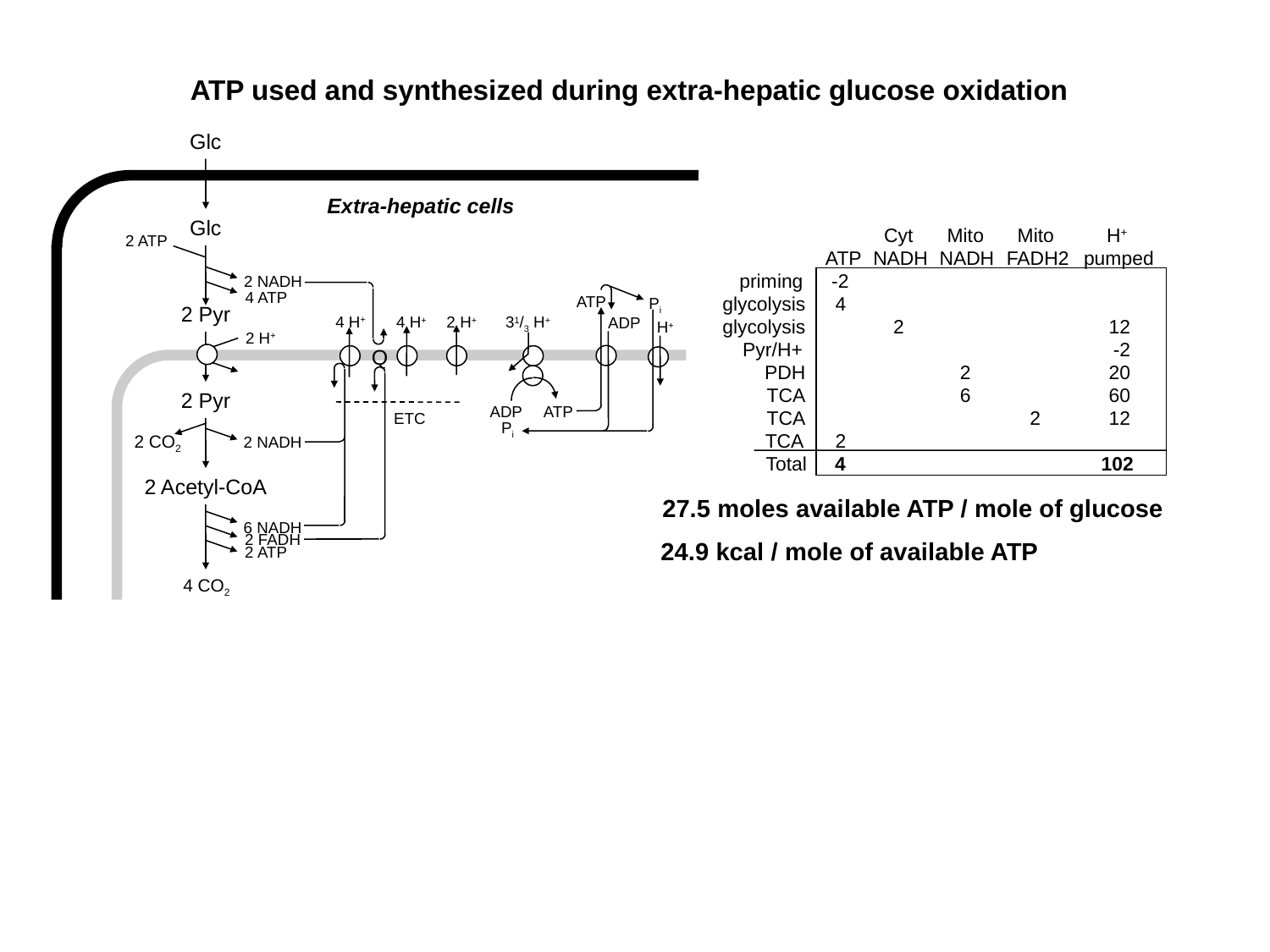

ATP used and synthesized during extra-hepatic glucose oxidation
Glc
Extra-hepatic cells
Glc
Cyt
Mito
Mito
H+
2 ATP
ATP
NADH
NADH
FADH2
pumped
2 NADH
priming
-2
4 ATP
ATP
Pi
glycolysis
4
2 Pyr
4 H+
4 H+
2 H+
31/3 H+
ADP
H+
glycolysis
2
12
2 H+
Q
Pyr/H+
-2
PDH
2
20
2 Pyr
TCA
6
60
ADP
ATP
ETC
TCA
2
12
Pi
2 CO2
2 NADH
TCA
2
Total
4
102
2 Acetyl-CoA
27.5 moles available ATP / mole of glucose
6 NADH
2 FADH
2 ATP
24.9 kcal / mole of available ATP
4 CO2
