# Supplementary material for: Effects of fructose-containing caloric sweeteners on resting energy expenditure and energy efficiency: a review of human trials
Source: Nutr Metab (Lond). 2013 Aug 13;10:54. doi: 10.1186/1743-7075-10-54 (PMC3751443; doi:10.1186/1743-7075-10-54)
Supplement: Additional file 2: Figure S2 — Energy cost of available ATP when fructose is converted into triglyceride palmitate in adipose tissue, and with subsequent release and oxidation of palmitate. The diagram depicts the key metabolic steps at which ATP, NADH and FADH2 are used or synthesized during fructose conversion into palmitic acid in hepatic cells, stored as triglyceride palmitate (with glycerol-3-P obtained from glycolysis) in adipose tissue, and subsequently released as palmitic acid to be oxidized in extra-hepatic cells. Calculations take into account that glycerol released from adipose tissue during lipolysis is reconverted into glucose in liver cells. The box at the bottom right summarizes the ATP used, synthesized, and available ATP made as in Additional file 1: Figure S1. Abbreviations: DHAP: dihydroxyacetone-P; Gly3P: glycerol-3-P; C16:0: palmitic acid; OA: oxaloacetate; Mal: malate; ACS: acyl-CoA synthetase; ACC: acetyl-CoA carboxylase; Cit lyase: citrate lyase; Pyr carb: pyruvate carboxylase; GlyK: glycerol kinase; EHC: extrahepatic cells; Ad: adipocytes; Li: liver; otherwise same as in Additional file 1: Figure S1. [file 1743-7075-10-54-S2.ppt]

## Slide 1
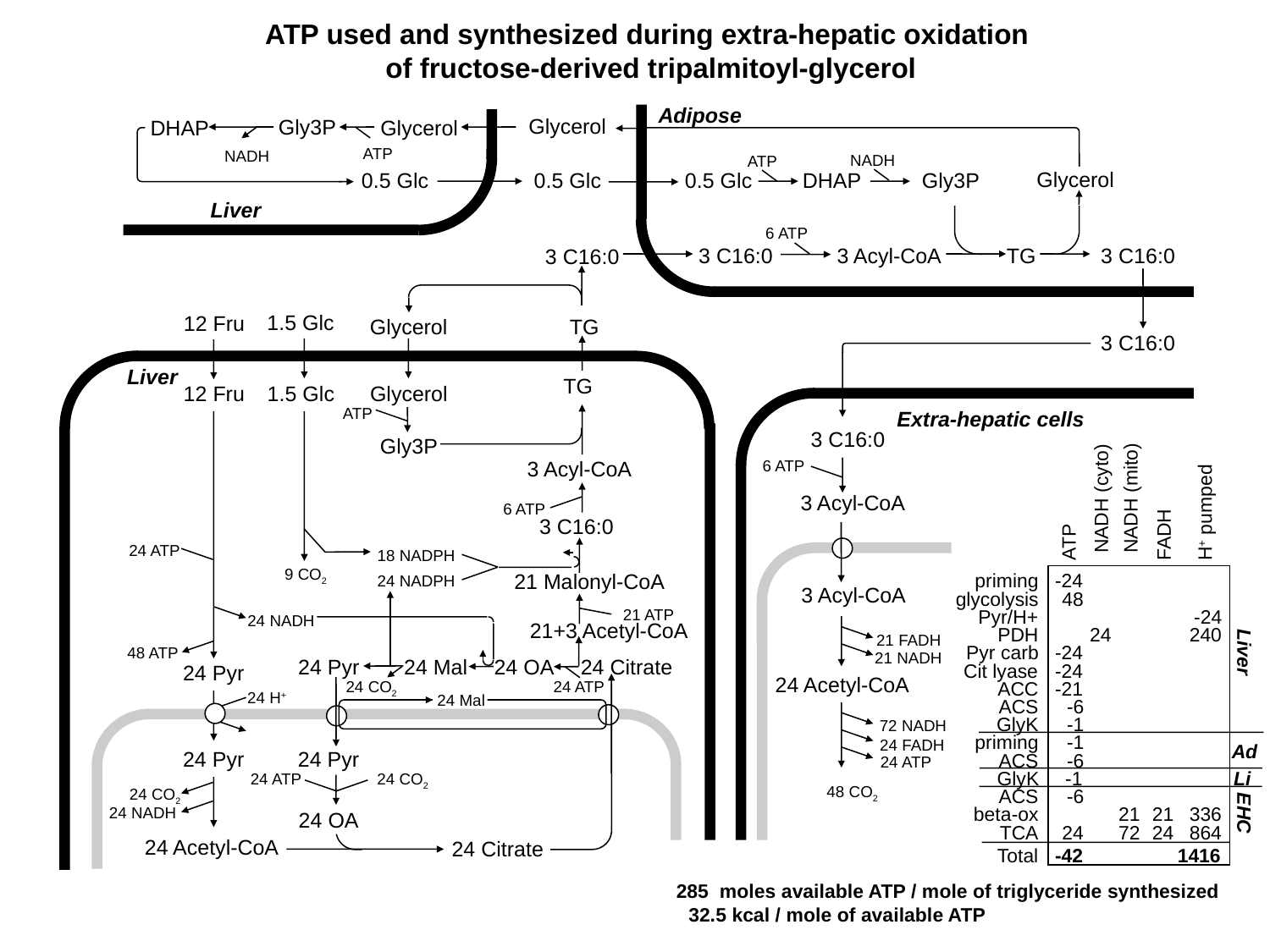

ATP used and synthesized during extra-hepatic oxidation
 of fructose-derived tripalmitoyl-glycerol
Adipose
Glycerol
Gly3P
Glycerol
DHAP
ATP
NADH
NADH
ATP
Glycerol
0.5 Glc
0.5 Glc
0.5 Glc
DHAP
Gly3P
Liver
6 ATP
3 C16:0
3 Acyl-CoA
TG
3 C16:0
3 C16:0
1.5 Glc
12 Fru
TG
Glycerol
3 C16:0
Liver
TG
12 Fru
1.5 Glc
Glycerol
ATP
Extra-hepatic cells
3 C16:0
Gly3P
3 Acyl-CoA
6 ATP
3 Acyl-CoA
NADH (mito)
NADH (cyto)
6 ATP
H+ pumped
3 C16:0
FADH
ATP
24 ATP
18 NADPH
9 CO2
21 Malonyl-CoA
24 NADPH
priming
-24
3 Acyl-CoA
glycolysis
48
21 ATP
24 NADH
Pyr/H+
-24
21+3 Acetyl-CoA
PDH
24
240
21 FADH
Liver
48 ATP
Pyr carb
-24
21 NADH
24 Pyr
24 Mal
24 OA
24 Citrate
24 Pyr
Cit lyase
-24
24 Acetyl-CoA
24 CO2
24 ATP
ACC
-21
24 H+
24 Mal
ACS
-6
72 NADH
GlyK
-1
24 FADH
priming
-1
Ad
24 Pyr
24 Pyr
24 ATP
ACS
-6
Li
24 ATP
24 CO2
GlyK
-1
48 CO2
24 CO2
ACS
-6
EHC
24 NADH
24 OA
beta-ox
21
21
336
TCA
24
72
24
864
24 Acetyl-CoA
24 Citrate
Total
-42
1416
285 moles available ATP / mole of triglyceride synthesized
32.5 kcal / mole of available ATP
